# Fostering learning engagement: the impact of different interpersonal relationships from the perspective of positive youth development

**DOI:** 10.3389/fpsyg.2024.1419588

**Published:** 2025-01-03

**Authors:** Mengjun Zhu, Xing’an Yao, Mansor Bin Abu Talib

**Affiliations:** ^1^Wellbeing Research Centre, Faculty of Social Sciences and Liberal Arts, UCSI University, Kuala Lumpur, Malaysia; ^2^School of Marxism, Nanjing Institute of Technology, Nanjing, China

**Keywords:** learning engagement, parent–child relationship, teacher-student relationship, peer relationship, intentional self-regulation

## Abstract

Learning engagement is a crucial factor affecting the quality of learning and holds significant value in promoting student development and enhancing teaching quality. By using time-lagged data from four schools and considering intentional self-regulation, this study integrates three types of relationships (parent–child, teacher-student, and peer relationships) into the same research framework to examine their impacts on learning engagement and the underlying mechanisms among high school students. The findings reveal that parent-child, teacher-student, and peer relationships all significantly positively affect high school students’ learning engagement. Intentional self-regulation plays a partial mediation effect between parent–child relationship and learning engagement, teacher-student relationship and learning engagement, along with peer relationship and learning engagement. The unique effect of peer relationship on learning engagement is significantly greater than that of teacher-student relationship but is not significantly greater than that of parent-child relationship. To better create a supportive synergy for enhancing students’ learning engagement, it is suggested that families and schools provide consistent learning support within their capabilities.

## 1 Introduction

In recent years, education has been a strategic priority in our country. With the progressive implementation of basic education curriculum reform, quality education has achieved remarkable results, yet there is still a gap in the requirement of “cultivating people through virtue.” Therefore, based on a series of supportive research, it is proposed to develop students’ core competencies from a top-level design perspective. Under the overall framework of core literacy, learning to learn is the most important core literacy (Lin, 2016). Enhancing students’ learning literacy level and effectively addressing the difficulties faced by educational development are critical factors in improving students’ learning engagement (Jia et al., 2018).

The focus of researchers has shifted towards positive psychological qualities, such as learning engagement, with the rise of positive psychology (D’Mello et al., 2017). Learning engagement refers to the sustained state of vigor, dedication, and absorption that students maintain when facing learning-related activities and contexts (Schaufeli et al., 2002). Concretely speaking, vigor implies students’ energetic engagement in learning, characterized by high levels of energy and mental resilience, encompassing a willingness to devote effort to their studies and the ability to persist in the face of challenges; Dedication means to an individual’s enthusiastic and proud commitment to learning, characterized by a pioneering spirit; Absorption refers to an individual’s complete focus on learning and their willingness to dedicate significant amounts of time to it (Carmona-Halty et al., 2019). Studies showed students academic achievements, academic exhaustion, and dropout intentions might be predicted by students’ learning engagement (Xiang et al., 2022; Bernardo et al., 2022; Wu et al., 2020). In addition, recent findings have shown that the state of learning engagement among Chinese students is concerning, with issues such as low learning efficiency and significant declines in engagement (Qiao et al., 2021). Hence, it is critical to enhance students’ level of learning engagement. Exploring the factors that influence learning engagement is essential not only for achieving better academic results but also for effectively supporting the development and improvement of adolescents’s learning abilities and preventing the risk of dropout.

## 2 Literature review

### 2.1 Interpersonal relationships and learning engagement

The environment in which an individual is situated plays a crucial role in influencing their learning engagement. According to Benson (2003), relationships across various contexts are significant determinants of learning engagement among children and adolescents. Interpersonal relationship is the direct psychological relationship formed by interaction and function between people. Good interpersonal relationships can improve individual health level and is the key factor to maintain the normal development of individual psychology. In contrast, disharmonious interpersonal relationship can trap individuals in self-doubt and self-denial, leading to a loss of motivation for learning and life goals (Zhang et al., 2020). Existing research has found that better interpersonal relationship, which brings more external social support, often lead to advancements in individual learning strategies. This not only facilitates improvements in student academic performance but also mitigates the impact of low self-regulated learning on individual psychological health (Davis and Humphrey, 2012).

Social support includes affirmations, approvals, and acceptance from multiple environments, such as family and school, allowing adolescents to experience a warm and caring atmosphere in healthy interpersonal relationship (Benson, 2002). The core ideas of positive youth development (PYD) emphasize relationship, viewing positive development as the result of intentional and meaningful relationship among adolescents (Benson, 2007). Moreover, the PYD perspective discovers adolescents from the viewpoint of “strengths and potentials,” highlighting the importance of the interplay between individuals and their surroundings in fostering positive development (Lerner et al., 2014). The ideal state of learning activities is to engage in meaningful learning with enthusiasm and positivity under the support of interpersonal relationship, thereby experiencing the realization of personal potential and self-worth (Yan et al., 2018).

Schools and families are the primary places and contexts for adolescent development. The teacher-student, peer, and parent–child relationships formed within these two major contexts are the main social relationship for children and adolescents (Zhao et al., 2021). Previous studies on the relation between interpersonal relationships and learning engagement either approached interpersonal relationship as a whole without differentiating between parent-child, teacher-student, and peer relationships (Collie et al., 2016) or focused on one of these relationship to investigate its influence on student learning engagement. These studies also found that all three types of interpersonal relationship can independently affect learning engagement (Shao and Kang, 2022; Thornberg et al., 2020; Sedláček and Šeďová, 2020). However, due to research limitations, previous research has not integrated parent–child, teacher-student, and peer relationships into the same research system to compare their effects on learning engagement. Comparing their impacts within the same research system is crucial for enhancing student learning engagement levels. As a result, the research hypotheses are as follows:

*H1*: Parent-child relationship can positively predict the high school student’s learning engagement.

*H2*: Teacher-student relationship can positively predict the high school student’s learning engagement.

*H3*: Peer relationship can positively predict the high school student’s learning engagement.

### 2.2 Intentional self-regulation as a mediator

Even in similar environments, individual development can vary (Dang et al., 2016). As research has progressed, scholars within the domain of positive youth development have found that good situational resources may activate positive developmental trajectories in adolescents, however, there might exist indirect pathways in these processes (Benson and Pittman, 2012). The relational development systems theory suggests that the interactions between the individual and his or her environments are the fundamental unit of human development, with intentional self-regulation being a key way individuals contribute to this interaction (Lerner et al., 2005). Previous research has classified self-regulation into organismic self-regulation and intentional self-regulation on the basis of the degree of conscious involvement. Gestsdóttir and Lerner (2008) further noted that during adolescence, intentional self-regulation under individual consciousness control matures and begins to become a significant factor.

Intentional self-regulation is the process by which individuals achieve positive self-development goals, mainly through selection, optimization, and compensation behaviors, aiming to optimally match situational demands, resources, and individual objectives (Gestsdóttir and Lerner, 2008). Research has demonstrated clearly that intentional self-regulation plays a significant role in the accurate prediction of academic performance and is the critical factor (Stefánsson et al., 2018) in burnout learning (Schunk and Ertmer, 2000), career aspirations (Napolitano et al., 2020), academic well-being (Chang et al., 2020) among middle school students. Additionally, the research has revealed that affirmation and support, companionship, and closeness from parents, peers, and teachers have a significant positive impact on the intentional self-regulation levels of middle school students (Zhou et al., 2021). Intentional self-regulation serves as a bridge between individuals and their environments, playing a crucial role in the adaptation process involving environmental variables (Lerner, 2006). Based on these research, the following research hypotheses are proposed:

*H4*: Intentional self-regulation would mediate the relation between parent-child relationship and learning engagement.

*H5*: Intentional self-regulation would mediate the relation between teacher-student relationship and learning engagement.

*H6*: Intentional self-regulation would mediate the relation between peer relationship and learning engagement.

Moreover, Considering the potential interactions among parent–child, teacher-student, and peer relationships, their effects on learning engagement may overlap if all three types of interpersonal relationships are included in the same structural equation model. Parent-child, teacher-student, and peer relationships may also have unique effects on learning engagement in addition to overlapping effects. The unique effect refers to the remaining predictive effect in a structural equation model that includes parent-child, teacher-student, and peer relationships, along with learning engagement, beyond the overlapping effects. Scholars generally believe that if the remaining predictive effect of an independent variable is not zero, then this variable has a unique effect on the dependent variable (McMahon et al., 2003). Thus, in the same model, the size of the unique effects can be compared to determine the influence of the three different interpersonal relationship on learning engagement. At this stage, direct comparisons of the unique effects of parent–child, teacher-student, and peer relationships on learning engagement are scarce, but research on the impact of home and school relationship on positive learning behaviors can provide references for this study. Research has found that as students age, their attachment to parents gradually decreases while their interactions with classmates increase, and simultaneously parent-child support decreases while peer support increases. The effect on learning engagement also changes, showing a state where peer support exceeds parent–child support on influencing learning engagement during this middle school stage (Zhuang et al., 2016). On the basis of the above studies, when comparing the unique effects of parent–child, teacher-student, and peer relationships on learning engagement, the present research attempts to consider the mediation effect of intentional self-regulation. Hence, this following research hypothesis is proposed:

*H7*: Considering the mediation effect of intentional self-regulation, the unique effect of peer relationship on learning engagement is significantly greater than that of parent-child or teacher-student relationships among high school students.

### 2.3 The present study

High school is a crucial stage that bridges earlier and later phases of school education, and it is a key period for developing and nurturing the literacy of “learning to learn” among students. High school students face a wide range of subjects, greater difficulty in knowledge, and the immense pressure of college entrance exams. They need to concentrate their efforts on learning activities, and experiencing meaning and value in their studies (Liu, 2016). Therefore, exploring the impact and mechanisms of parent–child, teacher-student, along with peer relationships on high school students’ learning engagement is of significant practical importance for enhancing the core core literacy levels of high school students and further improving the learning support system for high school students.

**Figure 1:**
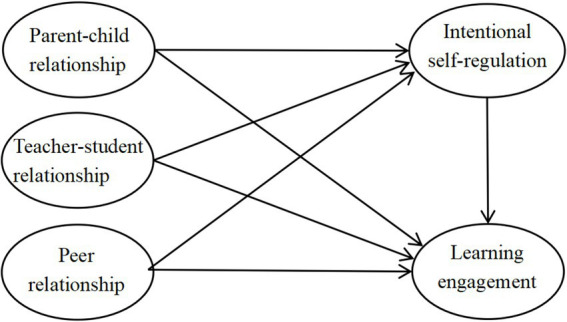
Conceptual framework of the study.

Moreover, although prior studies have mainly concentrated on the relation between one type of interpersonal relationships and learning engagement, less was understood about the relation between multiple types of interpersonal relationships and learning engagement, along with the roles of intentional self-regulation among them. Grounded in the aforementioned theories and empirical studies, the current research aims to explore the influence of parent–child, teacher-student, and peer relationships on learning engagement and their mechanisms among high school students. Integrating research hypotheses 1–7 forms a mediation model. Figure 1 shows the study’s hypothesis framework as follows:

## 3 Method

### 3.1 Participants

This study utilized a multi-stage stratified cluster sampling method, with the sample selection process divided into three stages: district division (2 out of 6 districts), school selection (4 schools), and class selection (12 classes). In 4 middle schools across central provinces of China, a sample of 600 survey were administered to students in grades 10–12 across 12 classes, with 540 valid questionnaires retrieved. After excluding questionnaires with incorrect answers to lie detection questions, patterned responses, or missing answers, the effective response rate was 90.00%. Demographic characteristics included age, gender (coded as male = 1; female = 2), grade (10th =1; 11th = 2; 12th = 3), and living area (1 = urban; 2 = rural). The sample consisted of individuals aged 15 to 18 years, with a mean age of 16.56 ± 0.90. There were 279 males (51.67%) and 261 females (48.33%). By grade, there were 181 students in 10th grade (33.52%), 184 in 11th grade (34.07%), and 175 in 12th grade (32.41%). Among them, 283 students (52.41%) were from urban areas and 257 students (47.59%) were from rural areas. Sample size estimation was performed using G*power 3.1 software (Faul et al., 2007), with *α* = 0.05, effect size *r* = 0.20, and aiming for 80% statistical power, at least 193 participants were required. The sample size used in this study met this requirement.

### 3.2 Measures

#### 3.2.1 Parent-child relationship

The middle school student’s parent–child relationship questionnaire designed by Wu et al. (2011) was employed. It consists of 26 items across four dimensions: understanding and communication (10 items, *α* = 0.919), harshness and interference (7 items, *α* = 0.903), fondness and respect (5 items, *α* = 0.897), and growth and tolerance (4 items, *α* = 0.847). Sample item is “When I talk, my parents listen patiently and attentively.” These dimensions encompass the cognitive, emotional, and behavioral facets of the parent–child relationship. Scoring is conducted using a 5-point Likert scale that extends from “1 = strongly disagree” to “5 = strongly agree.” Higher average scores across all items indicate better parent–child relationship status. In the current study, Cronbach’s alpha for this scale was 0.925.

#### 3.2.2 Teacher-student relationship

The Teacher-Student Relationship Scale developed by Zhang (2003) was adopted. This scale was created by referencing Pianta’s (1994) questionnaire and conducting interviews with teachers and students. It has 22 items and four dimensions: conflict (9 items, *α* = 0.921), attachment (5 items, α = 0.875), intimacy (4 items, α = 0.848), and avoidance (4 items, α = 0.891). Sample item is “I care about my teachers very much.” Scoring is conducted using a 5-point Likert scale, which progresses from “1 = strongly disagree” to “5 = strongly agree.” Higher average scores across all items represent a higher level of teacher-student relationship status. In the present study, Cronbach’s alpha for the scale was 0.931.

#### 3.2.3 Peer relationship

The Peer Relationship Scale for Children and Adolescents, designed by Guo (2003) and applicable to students aged 7–18, was used. This scale includes dimensions of friendship (5 items, *α* = 0.839), peer rejection (10 items, α = 0.907), and peer acceptance (7 items, α = 0.878). The scale comprises 22 items (e.g., “I focus on how other students see me.”) rated on a 4-point Likert scale ranging from “1 = strongly disagree” to “4 = strongly agree.” Items 11, 12, 15, 17, 19, 20, and 21 are scored negatively, while the remaining items are scored positively. Greater scores suggest a poorer peer relationship. In the present study, the Cronbach’s alpha for this scale was 0.922.

#### 3.2.4 Intentional self-regulation

The Selection, Optimization, and Compensation (SOC) Scale by Huang (2021) was adopted. This scale was designed by referencing Gestsdóttir and Lerner’s (2007) questionnaire. The scale comprises 17 items (e.g., “I always pursue goals one after the other.”). It includes three dimensions with 6 items for selection (*α* = 0.881), 5 items for optimization (α = 0.921), and 6 items for compensation (α = 0.922). The study utilized a 5-point Likert scale (1 = “very disagree,” 5 = “very agree”). Higher average scores across all items represent a higher level of intentional self-regulation. In this study, the Cronbach’s alpha coefficient for this scale was 0.935.

#### 3.2.5 Learning engagement

The Learning Engagement Scale, initially created by Schaufeli et al. (2002) and later updated by Fang et al. (2008), was utilized in this study. The scale comprises 17 items categorized into three dimensions: vigor (6 items, *α* = 0.878), dedication (5 items, *α* = 0.938), and absorption (6 items, *α* = 0.952). Sample item is “I feel energetic when I study.” The study utilized 7-point Likert scale, with 1 meaning “never” and 7 meaning “always.” Higher average scores indicate greater learning engagement. In the current study, the Cronbach’s alpha coefficient for this scale was 0.942.

#### 3.2.6 Covariates

In this study, age, gender, grade, and the number of siblings were controlled as covariates; this information was collected during the first data collection to prevent interference with the outcome variables. Previous research has shown that variables such as age, gender, grade, and the number of siblings are related to students’ learning engagement (e.g., Falbo, 2019; Santos et al., 2021; Zhong et al., 2023). Therefore, these demographic variables cannot be ignored when studying learning engagement among high school students (Liu et al., 2020).

### 3.3 Procedure

Research data was collected through self-administered questionnaires. Participant data were collected through an online survey, with teachers sending links to the informed consent form and survey questionnaire to class discussion groups. The aim of the research was introduced in the online questionnaire, and respondents could only submit their questionnaires after completing all items, minimizing the likelihood of accidentally skipping items. A participant could only submit one response. Given the potential for response bias in self-reported questionnaires, the anonymity and voluntary of the survey were disclosed to respondents. They were advised about the survey details and encouraged to answer all items truthfully, with assurances that the results would be kept confidential. Data were collected at two different times: demographic information and the three types of interpersonal relationship in Time 1, and intentional self-regulation and learning engagement in Time 2 (1 month later).

### 3.4 Data analytical plan

The statistical analysis of the data obtained would be performed using SPSS 25.0 and AMOS 24.0. To identify potential multivariate outliers, the Mahalanobis distance squared (MD^2^) method would be used (p1 and p2 < 0.001), and no outliers were removed. The suitability of the data for factor analysis would be assessed using the Kaiser-Meyer-Olkin (KMO) test and Bartlett’s test of sphericity. A KMO value of over 0.50 and Bartlett’s test significance of less than 0.01 (Hadia et al., 2016) would be required. As Hair et al. (2010) state, VIF values ranging from 1 to 5 represent multicollinearity that can be ignored. The Harman’s single factor test would be employed to appraise the common method bias (CMB). The CMB did not exist when a single factor with a small contribution was less than 50% only (Schwarz et al., 2017). The confirmatory factor analysis (CFA) for the latent variable loadings, concurrent validity as well as composite reliability were also considered. Pearson’s product–moment correlation test would be used to determine the relationship between these variables. Similarly, the structural equation model would be used to assess and adjust the model’s fitness and for the hypothesis test.

## 4 Results

### 4.1 Preliminary analyses

In this study, confirmatory factor analysis (CFA) was employed to examine the factor loading, convergent validity (CV) and component reliability (CR) of the five variables. The average variance extracted (AVE) values varied from 0.506 to 0.601 whereas CR varied from 0.803 to 0.819 and the factor load of the five latent variables ranged between 0.580 and 0.867, as Table 1 illustrates. The indicators have all been calculated the values recommended by Hair et al. (2019), implying that the five latent variable measuring methods enjoy good validity and reliability. As well, the square roots of the AVE values for each of the five latent variables were greater than the correlation coefficients between the variables, suggesting good discriminant validity among the five latent variables, according to the discriminant validity analysis of the five latent variables using AVE.

**Table 1:** Confirmatory factor analysis.

Variables	Confirmatory factor analysis			Discriminative validity				
	Factor loading	CR	AVE	PCR	TSR	PR	ISR	LE
Parent–child relationship	0.721 ~ 0.799	0.803	0.506	**0.711**				
Teacher-student relationship	0.701 ~ 0.807	0.819	0.531	0.383**	**0.729**			
Peer relationship	0.678 ~ 0.769	0.806	0.585	0.415**	0.431**	**0.765**		
Intentional self-regulation	0.580 ~ 0.854	0.817	0.601	0.494**	0.421**	0.521**	**0.775**	
Learning engagement	0.725 ~ 0.867	0.813	0.592	0.420**	0.434**	0.454**	0.480**	**0.769**

*p < 0.05, **p < 0.01, ***p < 0.001; bold values indicate the discriminant validity of the constructs. The same below.

Moreover, the means, standard deviations, and Pearson correlations of the main variables are presented in Supplementary Table S1. The results showed that the mean score for peer relationship (*M* = 2.194, *SD* = 0.560) was slightly below the midpoint of the scale, while parent–child relationship (*M* = 3.110, *SD* = 0.601), teacher-student relationship (*M* = 3.099, *SD* = 0.673), and intentional self-regulation (*M* = 3.050, *SD* = 0.751) showed medium level. In contrast, learning engagement (*M* = 4.606, *SD* = 1.111) was higher. Pearson correlations were used to analyze the relationship between each variable. Learning engagement among high school students can positively correlate with all three types of interpersonal relationships. Intentional self-regulation can significantly correlate with all three types of interpersonal relationships among high school students. And learning engagement was associated with intentional self-regulation among high school students. The correlation coefficients between each of the five variables were all less than 0.700, suggesting a good linear relationship between the five variables without multicollinearity issues, suitable for further analysis.

### 4.2 Direct effects of interpersonal relationship on learning engagement

Without considering intentional self-regulation, a model was constructed including parent–child, teacher-student, and peer relationships, along with learning engagement simultaneously to test H1, H2, and H3. Following the three-indicator presentation strategy recommended by Hair et al. (2010), the model fit was evaluated using the root mean square error of approximation (RMSEA), the Tucker-Lewis Index (TLI), and the standardized chi-square test. A model is considered to have a good fit if the RMSEA is less than 0.08, the TLI is above 0.90, and the standardized chi-square is less than 5 (Brown, 2006; Hair et al., 2010). The model accounted for the influence of gender and number of siblings. These control variables did not have a significant impact on interpersonal relationships and learning engagement. The fit indices indicate a good fit of the model to the data (χ2/df = 2.941, RMSEA = 0.060, TLI = 0.903), allowing further investigation of the relationship between latent variables to test the hypotheses.

**Figure 2:**
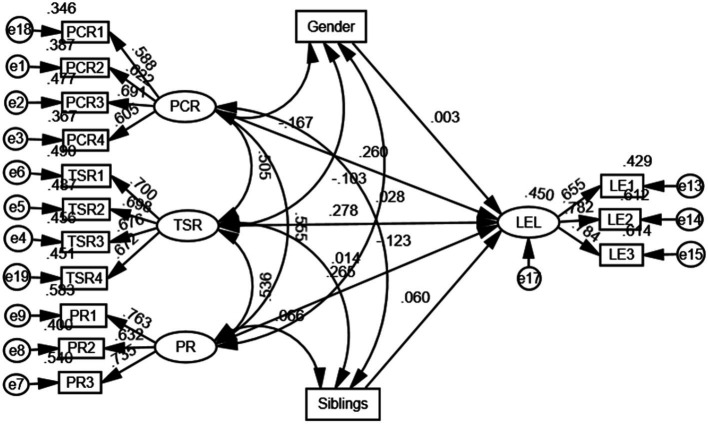
The direct effect model of interpersonal relationship on learning engagement.

The standardized regression coefficient of parent–child relationship on learning engagement (*β* = 0.258, *p* < 0.001) was positive and significantly different from zero, indicating that parent–child relationship can directly positively predict learning engagement, supporting H1. The standardized regression coefficient of teacher-student relationship on learning engagement (*β* = 0.277, *p* < 0.001) was positive and significantly different from zero, indicating that teacher-student relationship can directly positively predict learning engagement, supporting H2. The standardized regression coefficient of peer relationship on learning engagement (*β* = 0.270, *p* < 0.001) was positive and significantly different from zero, indicating that peer relationship can directly positively predict learning engagement, supporting H3 (see Figure 2).

### 4.3 Testing for mediation effects

**Figure 3:**
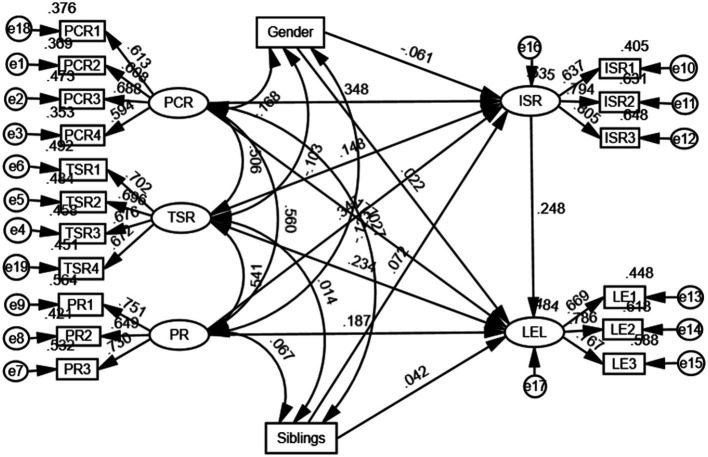
The impact model of interpersonal relationship and intentional self-regulation on learning engagement.

Considering intentional self-regulation as a the mediating variable and simultaneously examining the direct effects of parent-child, teacher-student, and peer relationships on learning engagement, hypotheses H1-H7 were tested. The model controlled for the impacts of gender and the number of siblings, which did not demonstrate significance for intentional self-regulation and learning engagement. The fit indices suggest that the model fits the data well (χ^2^/df = 2.834, RMSEA = 0.058, TLI = 0.903). In Figure 3, the standardized regression coefficient of parent–child relationship on learning engagement (*β* = 0.177, *p* < 0.05) was significantly different from zero, indicating that parent–child relationship can directly positively predict learning engagement, supporting H1. The standardized regression coefficient of teacher-student relationship on learning engagement (*β* = 0.234, *p* < 0.05) was significantly different from zero, indicating that teacher-student relationship can directly positively predict learning engagement, supporting H2. The standardized regression coefficient of peer relationship on learning engagement (*β* = 0.187, *p* < 0.05) was significantly different from zero, indicating that peer relationship can directly positively predict learning engagement, supporting H3.

To examine interpersonal relationships if indirectly affect learning engagement through intentional self-regulation. As shown in Table 2, the standardized regression coefficient of parent–child relationship on intentional self-regulation (*β* = 0.348, *p* < 0.05) is significant difference from zero, along with the standardized regression coefficient of intentional self-regulation on learning engagement (*β* = 0.248, *p* < 0.05) was also significantly different from zero. The mediation effect of intentional self-regulation was tested using Bootstrap resampling with 5,000 iterations. The bias-corrected (BC) 95% confidence interval is [0.028, 0.178], not containing zero, suggesting that intentional self-regulation mediates the effect, supporting H4. parent–child relationship have an indirect effect on learning engagement through intentional self-regulation, with a mediation effect of 0.086 (0.348*0.248).

**Table 2:** Structure path coefficient.

Path			Std. estimate	Estimate	S.E.	C.R.	*P*
Intentional self-regulation	←	Parent–child relationship	0.348	0.359	0.076	4.719	***
Intentional self-regulation	←	Teacher-student relationship	0.148	0.131	0.054	2.419	0.016
Intentional self-regulation	←	Peer relationship	0.347	0.351	0.073	4.819	***
Learning engagement	←	Parent–child relationship	0.177	0.278	0.119	2.341	0.019
Learning engagement	←	Teacher-student relationship	0.234	0.315	0.085	3.694	***
Learning engagement	←	Peer relationship	0.187	0.289	0.115	2.501	0.012
Learning engagement	←	Intentional self-regulation	0.248	0.378	0.122	3.103	0.002

Secondly, the standardized regression coefficient of teacher-student relationship on intentional self-regulation (*β* = 0.148, *p* < 0.01) was significantly different from zero. The mediation effect of intentional self-regulation was tested using Bootstrap resampling with 5,000 iterations. The bias-corrected (BC) 95% confidence interval is [0.011, 0.069], not containing zero, implying that intentional self-regulation mediates the effect, supporting H5. Teacher-student relationship has an indirect impact on learning engagement via intentional self-regulation, with a mediation effect of 0.037 (0.148*0.248).

Lastly, the standardized regression coefficient of peer relationship on intentional self-regulation (*β* = 0.347, *p* < 0.01) was significantly different from zero. The mediation effect of intentional self-regulation was tested using Bootstrap resampling with 5,000 iterations. The bias-corrected (BC) 95% confidence interval is [0.025, 0.158], not containing zero, meaning that intentional self-regulation mediates the effect, supporting H6. Peer relationship have an indirect effect on learning engagement through intentional self-regulation, with a mediation effect of 0.086 (0.347*0.248).

As seen Table 3, the unique impact of parent-child relationship on learning engagement is the sum of its direct and indirect effects, totaling 0.263 (0.177 + 0.086 = 0.263). Teacher-student relationship has a unique effect on learning engagement, which is equal to the sum of their direct and indirect effects, totaling 0.271 (0.232 + 0.037 = 0.271). Peer relationship has a unique effect on learning engagement, which is equal to sum of their direct and indirect effects, totaling 0.273 (0.187 + 0.086 = 0.273). Among the point estimates, peer relationship have the largest unique effect on learning engagement. Using Bootstrap resampling with 5,000 iterations, the differences in these unique effects were tested for significance. The results, with bias-corrected 95% confidence intervals of [−0.059, 0.069], which includes zero, indicate that the unique effect of parent–child relationship on learning engagement is not significantly less than that of peer relationship. The bias-corrected 95% confidence intervals of [−0.158, −0.006], which do not include zero, indicate that the unique effect of teacher-student relationship on learning engagement is significantly less than that of peer relationship. Thus, H7 is partially supported.

**Table 3:** Results for the conditional indirect effect model.

		Effect coefficient	Boot SE	Bootstrap 95% CI		*P*	Percentage of effect
				LLCI	ULCI		
Indirect effect	Parent–child relationship	0.086	0.038	0.028	0.178	0.012	32.700%
	Teacher-student relationship	0.037	0.016	0.011	0.069	0.019	13.653%
	Peer relationship	0.086	0.038	0.025	0.158	0.024	31.502%
Direct effect	Parent–child relationship	0.177	0.091	0.005	0.371	0.047	—
	Teacher-student relationship	0.234	0.066	0.086	0.365	0.015	—
	Peer relationship	0.187	0.085	0.028	0.379	0.011	—
Total effect	Parent–child relationship	0.263	0.079	0.124	0.423	0.004	—
	Teacher-student relationship	0.271	0.068	0.109	0.394	0.018	—
	Peer relationship	0.273	0.08	0.138	0.454	0.007	—
	Ind _(PCR → LE)_ vs. Ind _(TRS → LE)_	0.049	0.036	0.001	0.142	0.029	—
	Ind _(PCR → LE)_ vs. Ind _(PR → LE)_	0.000	0.031	−0.059	0.069	0.906	—
	Ind _(TRS → LE)_ vs. Ind _(PR → LE)_	−0.049	0.036	−0.158	−0.006	0.026	—

Dependent variable: learning engagement; Mediator: intentional self-regulation.

## 5 Discussion

From the perspective of family and school environments, the present research aimed to examine the impact of interpersonal relationships on learning engagement and the role of intentional self-regulation among high school students. The results indicate that parent–child, teacher-student, and peer relationships could significantly positively predict adolescents’ learning engagement. The direct predictive effect remained significant when intentional self-regulation was introduced as a mediator. Parent–child, teacher-student, and peer relationships could indirectly affect high school students’ learning engagement through intentional self-regulation.

### 5.1 Interpersonal relationships and learning engagement

The development of adolescent can be influenced by their relationship with significant others (Laird et al., 2018; Peterson et al., 2013). When they receive recognition and support from significant others, adolescent are more able to achieve a higher feelings of self-worth; when trust and support from significant others are lacking, psychological and behavioral problems may arise (Ryan and Deci, 2017). This study found that these three types of interpersonal relationships positively predicted learning engagement in different ways, echoing the positive youth development (PYD) theory. This theory considers good interpersonal relationships is a key factor for the healthy growth of teenagers, highlighting the significance of supportive interpersonal relationship in stimulating motivation and engagement in learning during adolescence (Lerner et al., 2014).

Parent-child relationship, by providing emotional support and a stable learning environment, lay the foundation for high school students’ learning engagement (Wang and Sheikh-Khalil, 2014). However, parents’ expectations and interventions may also increase students’ sense of pressure, especially during the academically demanding high school years (Kulakow et al., 2021). On the other hand, parents may have limited mastery of high school course content, which may not suffice to meet students’ needs in academic learning (Gonzalez-DeHass et al., 2005). Therefore, parent–child support should balance expectations and encouragement to avoid negatively impacting students’ learning engagement. Further findings indicated that the positive impact of teacher-student relationship highlighted the importance of teachers in motivating students, providing academic guidance, and emotional support (Lee et al., 2023). A caring and supportive teaching environment can significantly enhance students’ learning engagement and academic achievement. Good teacher-student relationship can promote high school students’ cognitive engagement, enhancing their interest and participation in learning. Nonetheless, the unequal distribution of educational resources may limit the opportunity for all students to engage in high-quality teacher-student interactions (Ansong et al., 2017). Meanwhile, peer relationships play a critical part in the socialization process of students, where support and positive modeling behaviors from peers can stimulate students’ interest and participation in learning (Wang and Eccles, 2012). However, negative interactions among peers, such as exclusion, bullying, and pressure, may lead to students’ avoidance behaviors toward learning, thus affecting their learning engagement (Gonida and Cortina, 2014).

Overall, these three types of interpersonal relationship collectively shape the learning environment and psychological state of high school students, having an indispensable impact on their learning engagement. These findings not only provide educators with diversified strategies to promote students’ learning engagement but also offer a new perspective on how adolescents develop and grow within different interpersonal relationship, especially in the context of current educational challenges (educational policy and environmental changes) and promoting comprehensive student development.

### 5.2 The mediating role of intentional self-regulation

Both the positive adolescent outlook on development and the system theory of relationship development point out that the influence of context on development outcome may be generated through individual self-system and individual behavior (Tian et al., 2015). In this study, intentional self-regulation plays a mediating role between interpersonal relationships and learning engagement of high school students. This study confirmed H4-H6. Based on relational development systems theory, the findings of this study highlight the complexity of individual-environment interactions and the central role of self-regulation in this process (Lerner et al., 2005). Individual development is achieved through dynamic interactions with the surrounding environment, where intentional self-regulation serves as a key mediator, enabling individuals to actively shape their own developmental trajectories (Lerner et al., 2014). Intentional self-regulation is a crucial aspect of adolescent development. Adolescents who can regulate their thoughts, emotions, and behaviors are more likely to succeed academically, have better relationships with peers and adults, and enjoy better mental health (Gestsdóttir and Lerner, 2007). Research has proved that although parental expectations can increase academic pressure, students can effectively balance these expectations with their own learning motivation through intentional self-regulation strategies (Stefánsson et al., 2018). These strategies include setting personal goals, optimizing resource use, and making behavioral adjustments, all of which enhance learning engagement. Positive teacher-student relationship provide essential emotional support and academic guidance, enabling students to more effectively utilize self-regulation strategies and thereby enhancing their learning engagement (Davis and Humphrey, 2012). Besides, with peer support and pressure, high school students can optimize their match with the environment through intentional self-regulation, maintaining or enhancing their engagement in learning (Zhou et al., 2021).

Furthermore, the findings indicate that with the mediating role of intentional self-regulation, the impact of peer relationships on learning engagement is significantly larger than that of teacher-student relationships. This outcome supports the findings of the study of Yu et al. (2023), who also determined that peer support becomes more important for keeping students in touch with the learning engagement as they age. This may be because during high school, students increasingly look for independence and autonomy, with peer influence and support becoming especially crucial during this learning phase (Zhuang et al., 2016). In spite of the unique effect of peer relationships on learning engagement is somewhat significant, the roles of parent–child and teacher-student relationships remain crucial factors that cannot be overlooked. These relationships provide different forms of support and resources that have a significant impact on both student motivation and emotional development. Therefore, strategies to promote student engagement in learning should take into account multiple interpersonal relationships and how these relationships can be optimized and adjusted through intentional self-regulation.

### 5.3 Implications

This study offers a new perspective for enhancing high school students’ learning engagement, with significant theoretical and practical implications. Theoretically, the present research contributes empirical evidence for positive youth development theory in the setting of high school students’ learning engagement, contributing to the existing literature. This study emphasizes the agency and regulatory abilities of individuals in their developmental process. Understanding how interpersonal relationship resources are optimized and utilized through self-regulation strategies. Secondly, by analyzing the unique effects of three types of interpersonal relationships on learning engagement, the current research provides a more detailed perspective to understand the influence of various types of interpersonal relationship on adolescent learning.

Practically, these findings are of significant importance to parents, school teachers, and policymakers. Firstly, confirming the central role of intentional self-regulation in promoting learning engagement means that schools and families should take measures to help adolescents develop and improve their self-regulation abilities. Secondly, given the significant impact of peer relationship on learning engagement during high school, schools should encourage the establishment of a positive and healthy peer culture. Additionally, as parents support their children’s learning, they should realize that the influence of peers increases as children grow, and therefore, need to adjust their support methods appropriately, offering necessary guidance and assistance while encouraging their children to think and solve problems independently. Lastly, education policymakers should consider the importance of interpersonal relationship in learning engagement, support schools in implementing student-centered teaching methods, promote positive interactions between teachers students, and peers, and through school-family cooperation, establish a comprehensive supporting learning climate for students.

### 5.4 Limitations and future directions

Despite the impacts mentioned above, this study still has some limitations. Firstly, this study only focused on self-reported surveys, which may have biases. To optimize this situation, subsequent research could consider integrating other research methods. Secondly, this study only explored the effect of intentional self-regulation in the process of how three kinds of interpersonal relationships affect learning engagement among high school students. In future research, more mediating variables will be considered in the model to reveal the complex process of how three types of interpersonal relationships affect learning engagement among adolescent. Lastly, the impact of three types of interpersonal relationships on student learning engagement varies at different learning stages. Limited by the scope of this study, it did not compare the effects of three kinds of interpersonal relationships at different learning stages. In future research, the influence of three kinds of interpersonal relationships on student learning engagement will be compared at different learning stages to clarify the important changes in interpersonal relationship in student learning growth.

## 6 Conclusion

In summary, the present research investigated the relation between parent–child, teacher-student, and peer relationships and high school students’ learning engagement from the perspective of positive youth development. All three types of interpersonal relationships had significant positive predictive impacts on learning engagement among high school students. Mediation analysis showed that intentional self-regulation could be an explanatory factor for the improvement of learning engagement by three types of interpersonal relationships among high school students. Additionally, considering the mediation effect of intentional self-regulation, the effect of peer relationship on learning engagement was significantly greater than that of teacher-student relationship among high school students.

## Data Availability

The datasets generated and/or analyzed during the current study are available from the corresponding author upon reasonable request.
