# Supplementary material for: Fostering learning engagement: the impact of different interpersonal relationships from the perspective of positive youth development
Source: Front Psychol. 2025 Jan 3;15:1419588. doi: 10.3389/fpsyg.2024.1419588 (PMC11738624; doi:10.3389/fpsyg.2024.1419588)
Supplement: Supplementary file 1 [file Table_1.docx]

Table 2

Descriptive Statistics and Correlations of Variables

Variables	1	2	3	4	5	6	7	8	9	10	11	12	13	14	15	16	17	18	19	20	21	22	23	24	25	26
1. gender	1																									
2. age	-0.059	1																								
3. grade	-0.037	0.762**	1																							
4. nos	-0.021	0.048	0.061	1																						
5. pcr	0.135**	-0.025	-0.059	0.013	1																					
6. uc	0.112**	-0.034	-0.071	0.002	0.782**	1																				
7. hi	0.099*	-0.031	-0.035	0.032	0.729**	0.273**	1																			
8. fr	0.080	-0.023	-0.039	-0.024	0.746**	0.409**	0.498**	1																		
9. gt	0.104*	0.044	-0.008	0.032	0.660**	0.395**	0.414**	0.354**	1																	
10. tsr	0.037	-0.068	-0.063	-0.001	0.383**	0.271**	0.282**	0.310**	0.279**	1																
11. con	0.054	-0.076	-0.083	-0.021	0.346**	0.254**	0.245**	0.292**	0.228**	0.855**	1															
12. att	-0.019	-0.034	-0.009	0.052	0.262**	0.162**	0.225**	0.199**	0.205**	0.742**	0.385**	1														
13. int	0.031	-0.063	-0.093*	0.006	0.289**	0.213**	0.203**	0.234**	0.208**	0.746**	0.587**	0.455**	1													
14. avo	0.032	-0.022	0.009	-0.028	0.267**	0.187**	0.188**	0.204**	0.226**	0.732**	0.433**	0.615**	0.354**	1												
15. pr	0.032	-0.003	-0.023	0.054	0.415**	0.317**	0.270**	0.311**	0.341**	0.431**	0.361**	0.346**	0.307**	0.315**	1											
16. fri	0.048	-0.042	-0.060	0.030	0.338**	0.246**	0.229**	0.275**	0.261**	0.318**	0.271**	0.252**	0.206**	0.247**	0.780**	1										
17. pr	0.039	0.039	0.029	0.052	0.337**	0.266**	0.218**	0.239**	0.278**	0.359**	0.292**	0.305**	0.266**	0.249**	0.846**	0.473**	1									
18. pa	-0.008	-0.031	-0.054	0.043	0.340**	0.257**	0.217**	0.256**	0.291**	0.365**	0.313**	0.272**	0.261**	0.274**	0.798**	0.590**	0.424**	1								
19. isr	0.076	0.008	-0.026	0.093*	0.494**	0.441**	0.298**	0.375**	0.302**	0.421**	0.359**	0.303**	0.322**	0.315**	0.521**	0.394**	0.451**	0.411**	1							
20. sel	0.076	-0.011	-0.018	0.124**	0.366**	0.309**	0.233**	0.271**	0.252**	0.350**	0.301**	0.237**	0.269**	0.272**	0.420**	0.334**	0.349**	0.339**	0.784**	1						
21. opt	0.078	-0.008	-0.052	0.053	0.449**	0.395**	0.296**	0.315**	0.277**	0.395**	0.352**	0.282**	0.289**	0.279**	0.459**	0.349**	0.404**	0.350**	0.840**	0.470**	1					
22. con	0.040	0.034	- .000	0.059	0.425**	0.399**	0.223**	0.351**	0.234**	0.319**	0.256**	0.244**	0.253**	0.243**	0.432**	0.310**	0.380**	0.344**	0.881**	0.516**	0.653**	1				
23. le	0.059	0.030	0.001	0.068	0.420**	0.380**	0.226**	0.324**	0.286**	0.434**	0.385**	0.335**	0.316**	.0285**	0.454**	0.363**	0.408**	0.324**	0.480**	0.338**	0.427**	0.435**	1			
24. vig	0.013	0.025	-0.008	0.039	0.296**	0.325**	0.129**	0.185**	0.184**	0.288**	0.257**	0.238**	0.180**	0.194**	0.352**	0.278**	0.315**	0.256**	0.425**	0.246**	0.397**	0.415**	0.801**	1		
25. ded	0.103*	0.012	0.008	0.084	0.381**	0.327**	0.215**	0.311**	0.259**	0.402**	0.363**	0.298**	0.293**	0.265**	0.431**	0.350**	0.382**	0.310**	0.442**	0.352**	0.372**	0.384**	0.835**	0.501**	1	
26. abs	0.034	0.036	0.001	0.047	0.374**	0.304**	0.218**	0.310**	0.269**	0.394**	0.342**	0.302**	0.311**	0.253**	0.360**	0.286**	0.329**	0.250**	0.346**	0.254**	0.310**	0.303**	0.870**	0.536**	0.605**	1
M	1.483	2.565	1.989	1.337	3.110	3.156	2.858	3.423	3.048	3.099	3.153	3.067	3.075	3.041	2.194	2.217	2.210	2.153	3.050	3.109	3.024	3.013	4.606	4.237	4.463	5.093
SD	0.500	0.900	0.813	0.525	0.611	0.759	0.824	0.928	0.863	0.673	0.817	0.845	0.866	0.960	0.560	0.714	0.665	0.700	0.751	0.811	0.954	0.937	1.111	1.176	1.447	1.377
Skewness	0.067	-0.088	0.020	1.222	-0.535	-0.157	0.054	-0.369	-0.031	-0.112	-0.214	-0.070	-0.045	0.109	0.017	0.080	0.074	0.268	-0.125	-0.231	0.134	0.044	-0.636	-0.165	-0.277	-1.020
Kurtosis	-2.003	-0.755	-1.485	0.476	0.185	-0.609	-0.659	-0.545	-0.590	-0.483	-0.641	-0.480	-0.561	-0.737	-0.582	-0.869	-0.809	-0.718	-0.407	-0.486	-0.693	-0.809	0.341	-0.762	-0.832	0.683

Note : Gender, Grade, and Number of siblings were a dummy variable, Gender: 0 = male and 1 = female; Grade: 1 = 10th, 2 =11th, 3= 12th; Number of siblings: 1 = 0, 2 = 1, 3 = 2, 4 = 3; nos = number of siblings; pcr = parent-child relationship; uc = understanding and communication; hi = harshness and interference; fr = fondness and respect; gt = growth and tolerance; tsr = teacher-student relationship; con = conflict; att = attachment; int = intimacy; avo = avoidance; pr = peer relationship; fri = friendship; pre = peer rejection; pa = peer acceptance; isr = intentional self-regulation; sel = selection; opt = optimization; com =compensation; *p < 0.05, **p < 0.01, ***p < 0.001.
